# Manifestation of relativistic effects in the chemical properties of nihonium and moscovium revealed by gas chromatography studies

**DOI:** 10.3389/fchem.2024.1474820

**Published:** 2024-09-23

**Authors:** A. Yakushev, J. Khuyagbaatar, Ch. E. Düllmann, M. Block, R. A. Cantemir, D. M. Cox, D. Dietzel, F. Giacoppo, Y. Hrabar, M. Iliaš, E. Jäger, J. Krier, D. Krupp, N. Kurz, L. Lens, S. Löchner, Ch. Mokry, P. Mošať, V. Pershina, S. Raeder, D. Rudolph, J. Runke, L. G. Sarmiento, B. Schausten, U. Scherer, P. Thörle-Pospiech, N. Trautmann, M. Wegrzecki, P. Wieczorek

**Affiliations:** ^1^ GSI Helmholtzzentrum für Schwerionenforschung, Darmstadt, Germany; ^2^ Helmholtz-Institut Mainz, Mainz, Germany; ^3^ Department of Chemistry, Johannes Gutenberg-Universität Mainz, Mainz, Germany; ^4^ Department of Physics, Lund University, Lund, Sweden; ^5^ Hochschule Mannheim, Mannheim, Germany; ^6^ Łukasiewicz - Instytut Mikroelektroniki i Fotoniki, Warsaw, Poland

**Keywords:** superheavy element chemistry, element 113–nihonium, element 115–moscovium, gas phase chromatography, recoil separators

## Abstract

Chemical reactivity of the superheavy elements nihonium (Nh, element 113) and moscovium (Mc, element 115) has been studied by the gas-solid chromatography method using a new combined chromatography and detection setup. The Mc isotope, ^288^Mc, was produced in the nuclear fusion reaction of ^48^Ca ions with ^243^Am targets at the GSI Helmholtzzentrum Darmstadt, Germany. After isolating ^288^Mc ions in the gas-filled separator TASCA, adsorption of ^288^Mc and its decay product ^284^Nh on silicon oxide and gold surfaces was investigated. As a result of this work, the values of the adsorption enthalpy of Nh and Mc on the silicon oxide surface were determined for the first time, $-∆H_{\text{ads}}^{{\text{SiO}}_{2}}\left(\text{Mc}\right)=54_{-5}^{+11}$ kJ/mol and $-∆H_{\text{ads}}^{{\text{SiO}}_{2}}\left(\text{Nh}\right)=58_{-3}^{+8}$ kJ/mol (68% c.i.). The obtained −Δ*H*_ads_ values are in good agreement with results of advanced relativistic calculations. Both elements, Nh and Mc, were shown to interact more weakly with the silicon oxide surface than their lighter homologues Tl and Bi, respectively. However, Nh and Mc turned out to be more reactive than the neighbouring closed-shell and quasi-closed-shell elements copernicium (Cn, element 112) and flerovium (Fl, element 114), respectively. The established trend is explained by the influence of strong relativistic effects on the valence atomic orbitals of these elements.

## 1 Introduction

### 1.1 Relativistic effects and periodicity trends in the superheavy element chemistry

Significant progress in the synthesis of the heaviest man-made chemical elements was achieved in the last decades resulting in the completion of the 7th row in the Periodic Table of the Elements (PTE) (Oganessian and Utyonkov, 2015). Due to decreasing nuclear fusion- and survival probabilities with increasing atomic number Z, the production rates of the superheavy elements (SHE) decrease rapidly, reaching a level of a single atom per month for oganesson (Og, element 118) (Oganessian and Utyonkov, 2015). The commonly used technique for SHE isolation is the kinematic separation in a recoil separator. The time between the production of a nuclear fusion reaction product in the target and its implantation as a recoil ion in the detection system is about 1 microsecond, allowing studies of nuclear decay properties of very short-lived isotopes. Experimental methods for chemical studies of SHE are more demanding and time-consuming, and therefore less efficient, especially for short-lived SHE isotopes. This makes studies of chemical properties more challenging (Türler and Pershina, 2013). Among the chemical techniques applied for SHE, a fast and efficient chemical separation can be achieved by gas-solid chromatography studies of volatile species (Türler and Pershina, 2013; Türler et al., 2015).

Chemical properties of yet unstudied SHE can be extrapolated based on the general law of periodicity, which connects the electronic structure of the elements with their position in the PTE. However, with increasing nuclear charge of the SHE, the influence of increasingly important relativistic effects on chemical properties has to be taken into account (Schwerdtfeger et al., 2020; Pyper, 2020; Pershina, 2019; Pershina, 2015). Apparently, the strong contraction of the s and p_1/2_ atomic orbitals (AO) and a large spin-orbit (SO) splitting of the electron shells with the orbital angular momentum quantum number l ≥ 1, can lead to properties different from those of their lighter homologues. Thus, reliable predictions of chemical properties of SHE become of extreme importance for the heaviest members of groups 12–18 (Schwerdtfeger et al., 2020; Pershina, 2019; Smits et al., 2024). Since the reactivity of atoms is generally connected to the energy level of their valence AO, relativistic calculations of the AO energy levels and of the first ionization potentials (IP) allow assessing the validity of trends within the groups of the PTE (Eliav et al., 2015; Borschevsky et al., 2015; Dzuba and Flambaum, 2016; Trombach et al., 2019). A strong relativistic stabilization of the spherical atomic orbitals with closed-shell 7s^2^ and quasi closed-shell 7p_1/2_^2^ electronic configurations, together with the large spin-orbit splitting within the 7p shell were shown to result in low AO energies and large IP values for Cn and Fl, respectively (Schwerdtfeger et al., 2020; Pershina, 2015; Eliav et al., 2015). Accordingly, these elements were predicted to be the least reactive members of groups 12 and 14 (Pitzer, 1975; Schwerdtfeger and Seth, 2002). These predictions were recently confirmed experimentally (Eichler et al., 2007; Eichler et al., 2010; Yakushev et al., 2014; Yakushev and Eichler, 2016; Yakushev et al., 2022).

The neighbouring odd-Z elements nihonium (Nh, element 113) and moscovium (Mc, element 115) are presently in the focus of theoretical studies. Since Nh and Mc possess one unpaired electron in the 7p_1/2_ or 7p_3/2_ subshells, respectively, higher AO energies and lower IP values than for Cn and Fl are predicted for Nh and Mc. This implies that the reactivity of Nh and Mc is higher compared with that of their closed-shell neighbours Cn and Fl. Therefore, chemical reactivity, i.e., the tendency to form chemical bonds, is predicted to be higher for Nh and Mc than for Cn and Fl (Schwerdtfeger et al., 2020; Pershina, 2015; Trombach et al., 2019). However, experimental data on the reactivity of Nh and Mc are required to validate the predicted local minimum at Fl. Indeed, Nh and Mc are expected to form chemical compounds, e.g., hydroxides, which should adsorb more strongly on surfaces than pure atoms (Pershina et al., 2009; Pershina and Iliaš, 2019). Recently, motivated by experimental developments (Yakushev et al., 2021), the interaction of Nh and Mc with Au and quartz surfaces was studied on the basis of periodic relativistic density functional theory (DFT) calculations (Pershina, 2016; Pershina et al., 2021). As a result, equal values of the adsorption energy/enthalpy (*E*_ads_ = −Δ*H*_ads_) on quartz, of 58 kJ/mol, were predicted for elemental Nh and Mc (Pershina et al., 2021). Significantly (by about 100 kJ/mol) higher *E*_ads_ values were predicted for the adsorption of these elements on the Au (111) surface in several theoretical works (Trombach et al., 2019; Pershina, 2018; Rusakov et al., 2013; Fox-Beyer and van Wüllen, 2012). The obtained *E*_ads_ values for Nh and Mc turned out to be significantly (by about 50–100 kJ/mol) lower than those of their lighter homologues, Tl and Bi, respectively, on both types of the surfaces, quartz and Au. This is due to the strong relativistic stabilization of the 7p_1/2_ AO with respect to the 6p_1/2_ AO (Pershina, 2018; Pershina, 2016; Pershina et al., 2021). Thus, all the available predictions agree that both Nh and Mc should be more reactive than Cn and Fl and should easily adsorb at room temperature on Au, and also adsorb on less reactive quartz surfaces (Ilias and Pershina, 2022).

### 1.2 Experimental challenges and methods

The gas chromatography method has proven being efficient for chemical studies of SHE compared to methods applied for chemical studies in the liquid phase (Türler and Pershina, 2013; Türler et al., 2015). However, the overall efficiency of this method is drastically dependent on the volatility and chemical reactivity of the species under study. In chemistry experiments with the lighter SHE rutherfordium (Rf, element 104) to bohrium (Bh, element 107), the chemical separation was achieved by the combination of the gas-jet transport with gas-solid chromatography (Türler and Pershina, 2013; Türler et al., 2015). A rather low overall efficiency of this multi-step technique was compensated by the relatively high production rates (Türler and Pershina, 2013). A significantly higher overall efficiency was achieved in the chemical study of hassium (Hs, element 108), which was separated in the form of the volatile compound HsO_4_ (Düllmann et al., 2002). This technique is based on the combination of gas-solid isothermal chromatography and thermochromatography with a detection system, i.e., by registration of the nuclear decay of a species adsorbed inside the chromatography column itself. The method was developed further during the past two decades, all along to the chemistry studies of the rather inert Cn and Fl (Eichler et al., 2007; Eichler et al., 2010; Yakushev et al., 2014; Yakushev and Eichler, 2016; Yakushev et al., 2022). The necessity of the spatial separation of the single volatile superheavy atoms from other volatile species that contain radionuclides with similar nuclear decay properties as the nuclide under study and that are created in vastly larger amounts in the form of unwanted side products of the nuclear reactions was recognised (Düllmann et al., 2005). This includes especially Rn isotopes and their daughters. This separation is nowadays routinely achieved by employing physical preseparation in an electromagnetic recoil separator (Yakushev et al., 2014; Yakushev et al., 2022; Wittwer et al., 2010).

Several offline and online studies of the adsorption of Tl species on quartz and gold surfaces were performed, in which a stronger interaction of Tl with Au than with quartz was found (Serov et al., 2013; Steinegger et al., 2016; Lens et al., 2018). In thermochromatography studies under dry and oxygen-free conditions, as well as in the presence of hydrogen, the deposition of Tl on gold was observed at a temperature of about 900°C and was assigned to elemental Tl (Serov et al., 2013). However, independent of the chemical composition in the gas phase, the deposition of Tl on quartz at a temperature of about 300°C was ascribed to be due to TlOH formation caused by a high reactivity of Tl towards hydroxyl groups expected to be present on the quartz surface (Serov et al., 2013). Whereas initial experimental studies with homologues are usually performed in chromatography columns made of bulk materials like quartz (fused silica) or Au metal foils, SHE experiments are often conducted in channels made of silicon radiation detectors with thin hetero-layers covering the detector surfaces. Thin silicon oxide (SiO_2_) or Au layers on silicon detectors are produced by oxidation or metal vapour deposition, respectively, and have poorly defined structures. These thin layers serve as reactive solid surface in the gas-solid chromatography process and are usually complex and differ from those used in most theoretical calculations on the adsorption of SHE.

For Cn and Fl, the focus of the chemical studies was on the interaction with Au surfaces (Eichler et al., 2007; Eichler et al., 2010; Yakushev et al., 2014; Yakushev and Eichler, 2016; Yakushev et al., 2022; Wittwer et al., 2010). In these experiments the adsorption enthalpy, $-∆H_{\text{ads}}^{\text{Au}}$ , could be measured in a range of approximately 65–35 kJ/mol. Despite the limited number of observed Cn and Fl atoms, both elements were conclusively shown to exhibit high volatility and a rather low reactivity towards Au, characterizing them as volatile metals. In one specific experiment, a SiO_2_-covered detector array was installed in front of the Au-covered ones. Neither Fl nor its decay product Cn adsorbed on this SiO_2_ surface at room temperature (Yakushev et al., 2022). The low reactivity of Fl, which was confirmed experimentally in several experiments (Eichler et al., 2010; Yakushev et al., 2014; Yakushev and Eichler, 2016; Yakushev et al., 2022), is ascribed to the strong relativistic stabilization of the closed 7p_1/2_ subshell (Schwerdtfeger et al., 2020; Pyper, 2020; Pershina, 2019). In contrast to rather inert Cn and Fl, which can be transported as gaseous species to a chromatography and detection setup (Eichler et al., 2010; Yakushev et al., 2014; Yakushev and Eichler, 2016; Yakushev et al., 2022), Nh and Mc might not. This makes experimental studies of these expectedly more reactive elements even more challenging (Yakushev et al., 2021). In fact, the transport of the presumably less volatile Nh to a detection setup was found to be inefficient (likely due to adsorption losses) through a tube made from a material as inert as polytetrafluoroethylene (PTFE) (Yakushev et al., 2021).

The longest-lived known Mc and Nh isotopes can be produced directly or as decay products in nuclear fusion reactions of ^48^Ca with ^243^Am and ^249^Bk targets, leading to the production of Mc and tennessine (Ts, element 117) isotopes, respectively (Oganessian and Utyonkov, 2015). The experimentally easiest access to Mc and Nh is via the nuclear fusion reaction ^243^Am (^48^Ca, 3n) ^288^Mc, which has a high cross section of approximately 10–20 pb (Oganessian et al., 2013; Rudolph et al., 2013; Gates et al., 2015; Oganessian et al., 2022; Oganessian et al., 2022b), similar to that for ^288^Fl produced in the reaction ^244^Pu (^48^Ca, 4n) ^288^Fl (Düllmann et al., 2010; Såmark-Roth et al., 2023), which was successfully used in chemical studies (Eichler et al., 2010; Yakushev et al., 2014; Yakushev and Eichler, 2016; Yakushev et al., 2022). The decay properties of the members of the ^288^Mc decay chain are rather well established based on more than two hundred observed decay chains (Oganessian et al., 2013; Rudolph et al., 2013; Gates et al., 2015; Oganessian et al., 2022; Oganessian et al., 2022b). The second member of the ^288^Mc decay chain, ^284^Nh, has a half-life of T_1/2_ = $0.90_{-0.06}^{+0.07}$ s (Oganessian et al., 2022b), similar to those of the isotopes ^287-289^Fl, which were in the focus of recent chemical studies (Yakushev et al., 2021; Eichler, 2013; Dmitriev et al., 2014; Aksenov et al., 2017). However, its directly produced shorter-lived precursor, ^288^Mc (T_1/2_ = $193_{-13}^{+15}$ ms) (Oganessian et al., 2022b), was out of reach due to the short lifetime that elapses before the Mc atoms reach the combined chromatography and detection system. The observation of several decay chains originating from ^284^Nh was claimed in early studies without physical pre-separation (Türler et al., 2015; Eichler, 2013; Dmitriev et al., 2014). The quality of the nuclear decay data in those works was limited by a rather high background in the α-particle spectra (Türler et al., 2015; Eichler, 2013; Dmitriev et al., 2014), which hampered the safe identification of decay chains (Türler et al., 2015; Yakushev et al., 2021). Two recent experiments employing preseparation were conducted behind the Dubna Gas-Filled Recoil Separator (DGFRS) (Akseneov et al., 2017), and behind the TransActinide Separator and Chemistry Apparatus (TASCA) (Yakushev et al., 2021). The low-background conditions led to a much higher sensitivity. However, no conclusive information on the chemical properties of Nh could be derived due to non-observation of ^284^Nh events in these experiments (Yakushev et al., 2021; Aksenov et al., 2017). The non-observation of Nh atoms in the experiments with preseparation was explained by losses due to the irreversible adsorption of Nh atoms on surfaces they encountered prior to reaching the detection setup, indicating a rather high chemical reactivity of the Nh atoms (Yakushev et al., 2021; Aksenov et al., 2017). Thus, no conclusive and confirmed results on the chemical properties of Nh were obtained in the previous studies. This called for further developments of the experimental setup. Gas chromatography studies of reactive species came within reach by employing the newly developed miniCOMPACT detector array (Yakushev et al., 2021), as demonstrated in adsorption studies of the lighter homologue of Mc, ^211^Bi^1^ and short-lived radioisotopes of the very reactive Fr (Götz et al., 2021). This improved detection system is promising for studies of Nh and is proven to be fast enough to observe a relevant fraction of all produced ^288^Mc.

## 2 Materials and methods

### 2.1 Experimental setup

**FIGURE 1:**
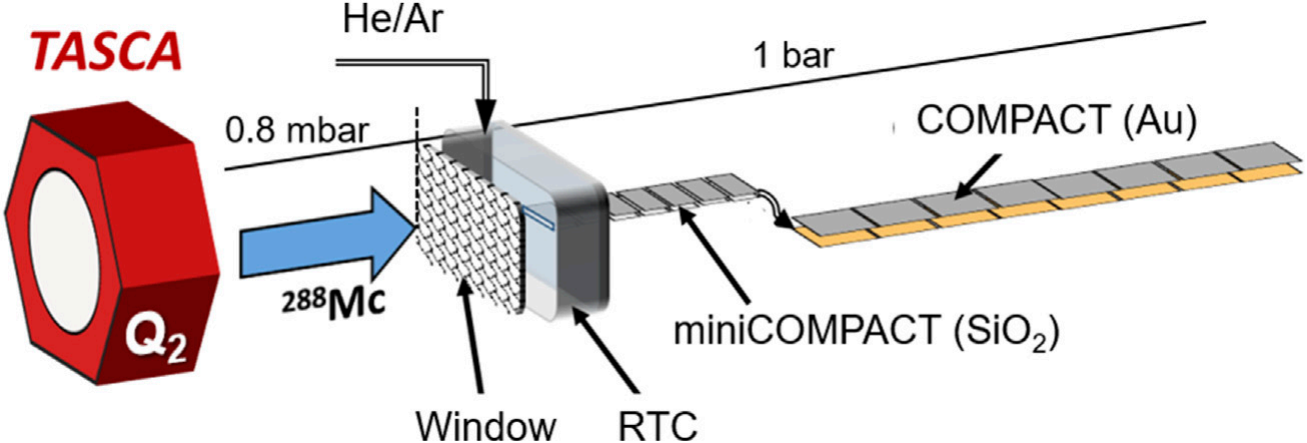
Experimental setup behind the second quadrupole (Q_2_) at the end of the TASCA separator. Recoiling ^288^Mc produced in the nuclear reaction were separated with TASCA and entered the Recoil Transfer Chamber (RTC) behind TASCA through a Mylar window. They were thermalized inside the RTC, the inner surface of which was covered with a Teflon™ layer and flushed out with a He/Ar gas mixture to the gas chromatography setup. The first gas chromatography and detection channel, miniCOMPACT, is connected directly to the RTC exit and covered with a SiO_2_ layer. The second detector array COMPACT has an Au layer on the detector surface. Both detector arrays were operated at room temperature.

The nuclear reaction ^243^Am (^48^Ca, 3n)^288^Mc, utilized for the discovery of element 115, Mc (Oganessian and Utyonkov, 2015), and for the detailed studies of the decay properties of the long α-decay chain starting from the isotope ^288^Mc (Oganessian et al., 2013; Rudolph et al., 2013; Gates et al., 2015; Oganessian et al., 2022; Oganessian et al., 2022b), was also used for the present chemical studies of Mc and Nh. The gas-filled recoil separator TASCA (Semchenkov et al., 2008) was used to separate products of the fusion-evaporation reaction from the intense primary beam and from the unwanted products of multi-nucleon transfer reactions. The chemical reactivity of Mc and Nh was investigated applying the gas-solid chromatography method in the isothermal regime at room temperature (20°C) (Türler and Pershina, 2013; Türler et al., 2015) via adsorption studies on two surface types, SiO_2_ and Au. The gas chromatography setup behind TASCA, as shown in Figure 1, was similar to that described in the first experiment on Nh chemistry at TASCA (Yakushev et al., 2021), except for the connecting tube between the Recoil Transfer Chamber (RTC) and the first detector array. The distance from the RTC gas volume to the first detector element was reduced to about 1 mm.

**FIGURE 2:**
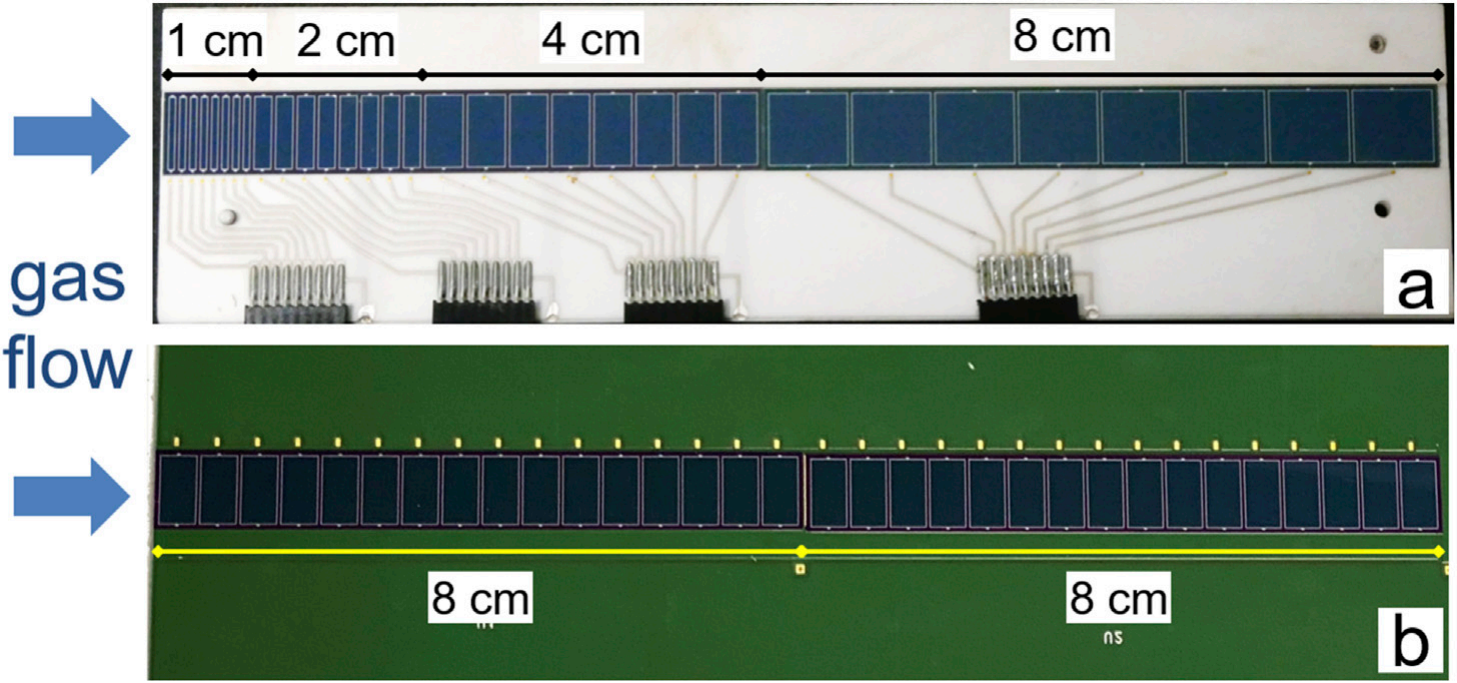
The open miniCOMPACT detector arrays. Only one of two detector panels forming the narrow channel is shown, as used in the first **(A)** and second experimental run **(B)**. The direction of the gas flow through the detector channel is indicated with arrows.

A pulsed (5 ms beam-on, 15 ms beam-off) ^48^Ca^10+^ ion beam was provided by the UNIversal Linear ACcelerator (UNILAC) at GSI Darmstadt with a typical beam intensity of 5 10^12^ s^−1^. The target wheel contained four ^243^Am_2_O_3_ targets and rotated with a speed of 2000 revolutions per second, synchronized with beam pulses (Jäger et al., 2014). The ^243^Am targets with an average thickness of 0.83(1) mg/cm^2^ were electro-deposited on 2.2(1) μm Ti foils (Runke et al., 2014). The beam energy in the middle of the ^243^Am targets was 243.0(2) MeV. In total, 1.2(1) 10^19^ ^48^Ca ions impinged on the targets during two experimental runs of 15 and 26 days duration, respectively. The evaporation residues were guided through the magnetic recoil separator TASCA with one dipole (D) and two quadrupole (Q) magnets in DQ_1_Q_2_ configuration, operated in the High-Transmission Mode (Semchenkov et al., 2008). TASCA was filled with He at a pressure of *p*_He_ = 0.8 mbar and was set to a magnetic rigidity of *B·ρ* = 2.21 Tm (Rudolph et al., 2013; Khuyagbaatar et al., 2012) to center ^288^Mc recoils in the TASCA focal plane. The nominal transmission efficiency in TASCA to focus the recoiling ^288^Mc ions into the RTC window area is about 40% (Yakushev et al., 2021). Products recoiling from the target and separated in TASCA according to their magnetic rigidities were thermalized in the RTC in a 1:1 gas mixture of He and Ar at 1 bar, similarly to the first Nh study at TASCA (Yakushev et al., 2021). Due to a large number of collisions with gas atoms, the isolated ions lose their kinetic energy, and their charge state is decreased accordingly to +1 or +2. The thermalized ions can be neutralized in gas by recombination or collisions with gas impurities or with any surface. The gas was circulated in a closed loop by a membrane pump at a flow rate of 2.9 L/min. The rare gases, He (99.9999%) and Ar (99.999%), were purified further by passing cartridges MC50-902FV and MC400-902FV (SAES™). These cartridges had internal particle filters, which efficiently remove aerosol particles with sizes down to 3 nm diameter and reduced the water and oxygen content to a level of about 1 ppm. The most critical impurity, water, was monitored by the dew point transmitter Pura (Michell Instruments). The isolated nuclear reaction products were flushed into the gas chromatography and detection setup. Two different detector channels were used, namely, a miniCOMPACT detector (Yakushev et al., 2021; Götz et al., 2021), followed by a COMPACT detector, used in the previous Fl chemistry studies at TASCA (Yakushev et al., 2014; Yakushev et al., 2022). The miniCOMPACT detector array was directly connected to the RTC via an exit slit with a cross section of (10.0 × 0.5) mm^2^, fitting to the cross section of the miniCOMPACT channel, and allowing for an effective transport and detection of less-volatile species. As Nh and Mc should interact more strongly with gold than with quartz (Pershina et al., 2021), the silicon photodiodes of the miniCOMPACT detector array were covered with a 30–50 nm-thick SiO_2_ layer. The photodiodes of the COMPACT detector array were covered with a 30 to 50 nm-thick Au layer. A 30-cm long capillary made of PTFE with an inner diameter of 2 mm connected the Au-covered COMPACT detector array to the miniCOMPACT array. Both detectors consisted of two panels, each divided into 32 detector elements. The opposite detector panels (top and bottom) were mounted with the active detector surfaces facing each other, and thus, forming a narrow gas channel with a distance between the opposite panels of 0.6 mm or 0.8 mm for the COMPACT array and the miniCOMPACT array, respectively. Each detector panel of the miniCOMPACT and COMPACT detectors consists of 32 Positive-Intrinsic-Negative (PIN) diodes mounted in a row (Wegrzecki et al., 2013). They registered α particles and fission fragments emitted by nuclear decays occurring inside the detector channel. Two different segmentation geometries of the miniCOMPACT detector arrays were used in the first and the second run. In the first run, a 15-cm long miniCOMPACT array had differently-sized detector elements mounted on a ceramic printed circuit board (PCB) as shown in Figure 2A. Each detector panel consisted of two silicon chips, which were 7 cm and 8 cm long. The first 7-cm long silicon chip was divided into three eight-element groups. They were 1, 2, and 4 cm long. The following 8-cm long chip had eight equal detector elements. In the second run, a 16-cm long miniCOMPACT detector array was used. The panels of the second detector consisted of two equal, 8-cm long silicon chips, each divided into sixteen detector elements, and were mounted on a multilayer FR4 PCB (flame retarding printed circuit board) as shown in Figure 2B.

### 2.2 Method of calculation

Theoretical calculations accompany our experimental study. We have calculated electronic energies of gas-phase reactions that single metal atoms (M, where M = Bi and Mc) and singly-charged metal ions (M+) could undergo in the recoil chamber. The aim was to investigate a potential reactivity of Bi and Mc species towards water and oxygen, which are present as gas impurities at the trace level.

Our considered systems are SHE, which can only be treated appropriately when relativistic effects are explicitly taken into account in the calculations. A well-proven method for such work is the Density Functional Theory (DFT) approach (Hohenberg and Kohn, 1964). The theory underlying is the DFT is the self-consistent Kohn–Sham approach (Kohn and Sham, 1965). Kohn-Sham DFT is a first-principles computational method to predict accurately chemical properties and to analyse them in simple chemical terms. Specifically, the Kohn-Sham approach in general delivers correct electron densities and related properties, as well as the total energy, but implies a one-electron representation of the many-electron systems. Although the precise exchange-correlation (XC) functional is unknown, by using appropriate XC functionals, binding energies may typically be determined with a precision of a few kJ/mol (Te Velde et al., 2001). DFT keeps at all levels of approximation the appealing one-electron molecular orbital (MO) view on chemical reactions and properties. The computed orbitals are suitable for typical MO-theoretical analyses and interpretations. The Kohn-Sham method also effectively incorporates all correlation effects. The calculations of electronic structures of the molecules of interest were performed using the Density Functional Theory approach implemented in the AMS ADF software (SCM, 2024). The AMS electronic structure approach uses the zeroth order regular approximation (ZORA) two-component Hamiltonian incorporating both scalar relativistic (SR) and SO relativistic effects. Although the regular approximation is not gauge invariant at any order, the scaled ZORA energy is exactly gauge invariant for hydrogen ions. The scaled ZORA approach, as it was used in our ADF calculations, is nearly gauge-invariant for many-electron systems, as explained in detail in Ref. (Van Lenthe et al., 1994). Briefly, the scaled ZORA equation differs from the original ZORA equation by the denominator to introduce a scaling of the ZORA energy. The calculated scaled-ZORA total energies prove to be very close to the fully relativistic energies for atoms. For all-electron calculations, the original ZORA total energy cannot be used as it suffers from gauge invariance problems that are particularly serious for deep-core states. The scaled ZORA total energy solved this problem, delivering results within “chemical” accuracy. The same holds for two-component calculations with the SO ZORA Hamiltonian. The spin-polarized non-collinear approach, also called as Kramers unrestricted approach, was applied. The basis sets are a combination of numerical AOs and Slater-type orbitals (STOs). The used basis sets are of the Triple Zeta plus Double Polarization (TZ2P) size. These basis sets are well saturated and describe the polarization of the electron density of an atom in molecules. Since correlation effects are also very important for SHE systems and can contribute up to the 50% of the binding energy, those are taken into account by using XC functionals. We utilized the BP86 (Becke, 1988) exchange-correlation functional. The geometry optimizations of studied molecules were carried out with the SO ZORA Hamiltonian, ensuring that both SR and SO relativistic effects were reflected in the optimized molecular structures.

## 3 Results

### 3.1 Observation of decay chains from ^288^Mc and ^284^Nh

**FIGURE 3:**
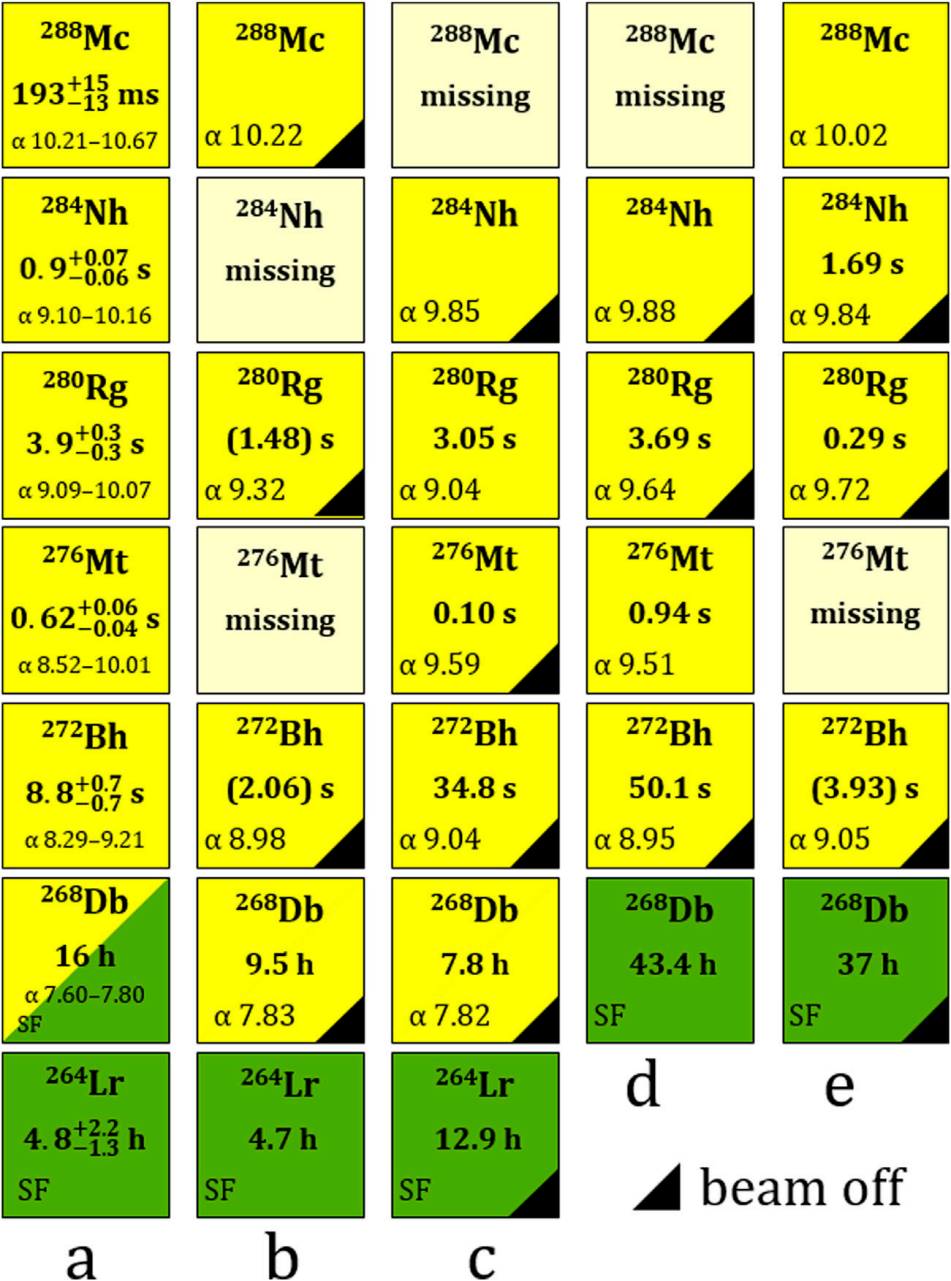
The decay properties of the ^288^Mc-decay chain members. **(A)** The presently known decay properties along the long decay chain from ^288^Mc are shown for comparison (Oganessian et al., 2022). **(B–E)** Four representative decay chains (#2, #8, #5, #12 in Table 1) out of eighteen observed in our experiments are shown. Color coding: α decay (yellow) and spontaneous fission (green). Half-lives **(A)** and correlation times **(B–E)** are indicated for decay chain members. The total time difference for decay chain members following missing ones are given in parentheses. The α-particle energies are given in mega electron volts (MeV). The black triangles represent the decays observed during beam-off periods.

A search for position- and time-correlated nuclear decay chains was performed, to identify multiple genetically-linked members of the well-known long ^288^Mc decay chain, decaying by emission of α-particles within the energy interval (8.0–10.5) MeV and within a 200-s time interval. This search resulted in the finding of eighteen decay chains, seven in the first run and eleven in the second run. The decay chains were assigned to ^288^Mc or to ^284^Nh in accordance with their known decay properties (Oganessian et al., 2022; Oganessian et al., 2022b; Forsberg et al., 2016; Rudolph, 2022). As usual for such an experiment, where an atom is adsorbed on a detector surface inside a gas chromatography channel, α particles or fission fragments have to penetrate several inactive layers. They deposit a fraction of their kinetic energy in a SiO_2_ or Au layer on top of the detector surface, and then in an inactive silicon layer on top of the diode. In addition, if the particles cross the detector channel, they experience further energy loss in the gas. These energy losses depend strongly on the angle to the surface, at which the particle is emitted. Thus, the energy distributions of registered α particles and spontaneous fission (SF) fragments have usually tailing towards low energies in contrast to the orresponding energy distributions registered in physics experiments, where the ions are implanted into a silicon detector. The energy calibration of the detectors was performed using a ^227^Ac source emanating ^219^Rn, and was linearly extrapolated up to the fission fragment energies. Independently, the search for high-energy events with an energy of E_SF_ > 20 MeV was performed on a time scale of hours to days, to identify candidates for long-lived decay chain members terminating the decay chains by SF. In total, twenty-one candidate SF events were identified in the two runs. Sixteen SF events, twelve with two fragments and four with one fragment, occurred at the same detector positions, where chains with multiple α-decaying members were also detected, several hours before the SF events. These position-correlated SF events were assigned to the SF decay of ^268^Db or ^264^Lr (Oganessian et al., 2022). For two out of the eighteen registered decay chains, no terminating SF events were found, presumably due to a several-hour long problem with the data acquisition system in the period following the detection of these two chains. The decay parameters, energies and time intervals of the members of all observed decay chains are listed in Table 1. Figure 3 shows four representative examples (b–e) of observed chains, along with the reference decay chain (a).

**TABLE 1:** Energies of α particles (E_α_) and SF (E_SF_) fragments^a^ and time intervals (Δt) of the observed decay chain members and fission events without α-decaying precursors. The total time difference for decay chain members following missing ones are given in parentheses.

Chain number	^288^Mc E_α_, MeV	^284^Nh E_α_, MeV Δt_1_, s	^280^Rg E_α_, MeV Δt_2_, s	^276^Mt E_α_, MeV Δt_3_, s	^272^Bh E_α_, MeV Δt_4_, s	^268^Db E_α/SF_, MeV Δt_5_, h	^264^Lr E_SF_, MeV Δt_6_, h
1	**10.33** ^b^ −	**9.84** ^c^ 0.646	missing	**9.51** (22.86)	missing	**85 + n.d.** ^d^ (40.6)	
2	**10.22** −	missing	**9.32** (1.48)	missing	**8.98** (2.06)	**7.83** 9.5	**74 + 70** 4.7
3	missing	**9.25** −	**9.71** 3.33	9.76 0.515	**9.05** 7.43	**>64+>66** ^e^ 13.8	
4	missing	**9.79** −	9.70 9.22	**8.95** 0.289	**8.97** 3.27	**7.69** 24.2	98 + 110 6.4
5	missing	**9.88** −	**9.64** 3.69	9.51 0.938	**8.95** 50.13	93 + 116 43.4	
6	missing	**9.57** −	**9.69** 3.19	**9.56** 0.813	**8.91** 8.94	**112 + n.d** ^d^ 64.8	
7	missing	**9.47** −	**9.70** 0.813	**0.76** ^f^ 0.125	missing	**7.77** (14.3)	**117 + 66** 13.3
8	missing	**9.85** −	**9.04** 3.05	**9.59** 0.105	**9.04** 34.84	**7.82** 7.8	**49 + n.d** 12.9
9	missing	**9.86** −	9.53 0.253	**9.27** 0.125	**8.82** 8.39	**91 + 93** ^g^ 19.1	
10	**10.38** −	**9.44** 0.318	9.84 19.38	**9.47** 2.05	missing	n.d.^h^	
11	missing	**9.98** −	**9.72** 8.41	**9.45** 0.285	missing	n.d.^h^	
12	10.02 −	**9.84** 1.69	**9.72** 0.293	missing	**9.05** (3.93)	**116 + 86** ^g^ 37	
13	missing	9.92 −	**9.78** 1.93	**9.54** 0.084	8.07 5.76	**111 + 83** ^g^ 81.2	
14	missing	9.96 −	missing	**9.62** (92.68)	8.58 13.46	**83 + 89** ^g^ 30.1	
15	missing	**9.60** −	**9.68** 0.937	**9.44** 1.12	**9.06** 5.18	70 + 75^i^ 8.9	
16	missing	**9.65** −	**9.61** 6.74	**9.24** 0.034	**8.84** 6.42	**87 + 103** ^i^ 43.7	
17	missing	**9.63** −	**9.58** 16.91	**9.37** 0.432	missing	**11 + 28** ^i^ 21.9	
18	missing	**9.75**	missing	**9.63** (0.551)	**9.08** 5.44	77 + n.d.^i^ ^,^ ^j^ 31.8	
Fission events without α-decaying precursors							
SF 1		SF 2		SF 3		SF 4	SF 5
105 + n.d		104 + 113		91 + 70		99 + 93	99 + 22

^a^ The SF fragment energy values were not corrected for the pulse height defect.

^b^ The energy values of decay chain members, where decay occurred during the beam-off periods, are given in bold.

^c^ The decays from decay chain members starting with ^284^Nh occurred 4 mm downstream to the decay from ^288^Mc.

^d^ One SF fragment was not detected (n.d.) due to the not working opposite detector strip.

^e^ This event occurred in the second Au-covered COMPACT detector. The full SF fragment energy could not be detected due to a high amplification gain of the preamps.

^f^ A small energy signal was detected. This can be explained by α particle hitting mainly the inactive area of the detector.

^g^ No candidates for α decay of ^268^Db.

^h^ SF events were not detected due to a technical problem with the data acquisition system lasted for several hours.

^i^ Several candidates for the α decay were found. No unambiguous conclusion on α decay of ^268^Db could be made.

^j^ All signals were registered on the same detector side pointing at the deposition on the inactive surface of the opposite detector side.

**FIGURE 4:**
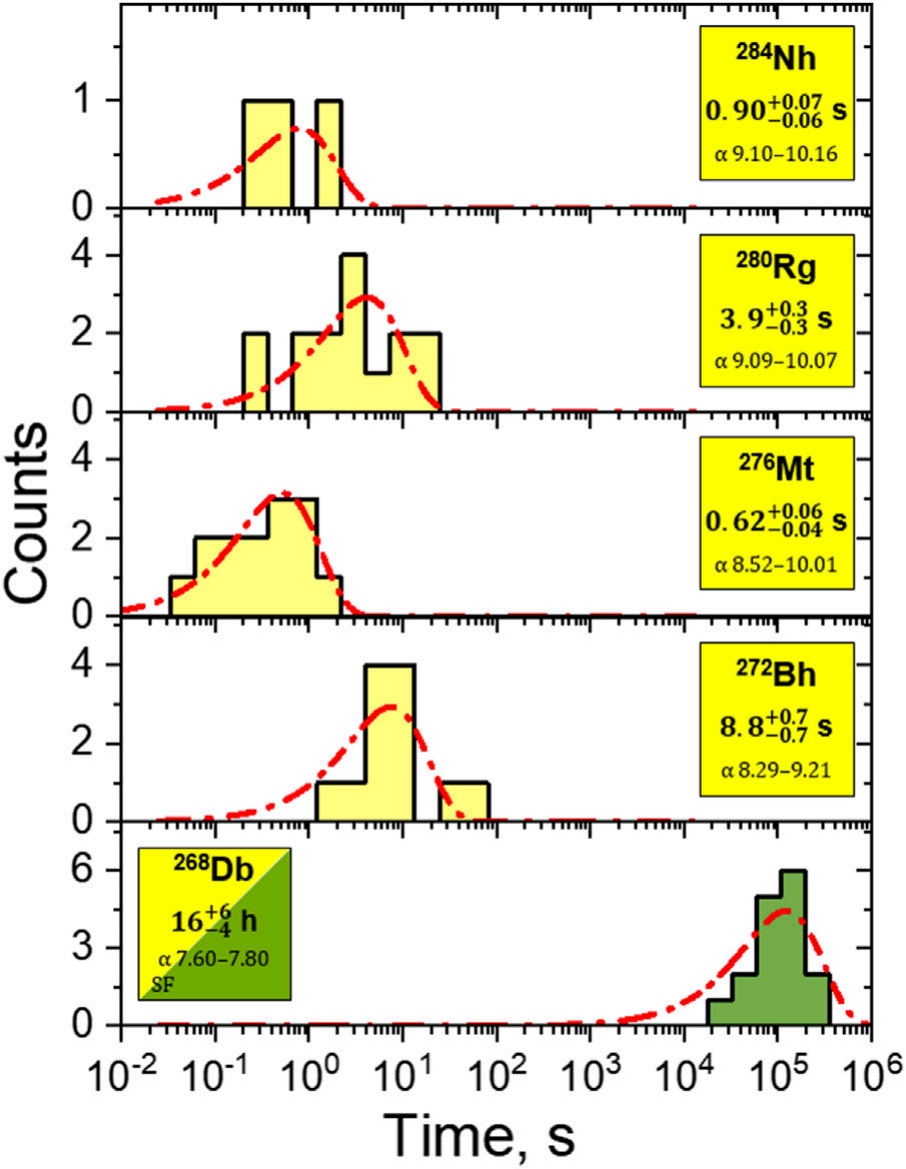
The lifetime distributions of the observed decay chain members ^284^Nh, ^280^Rg, ^276^Mt, ^272^Bh, and ^268^Db. The experimental lifetime distributions are shown for ^284^Nh, ^280^Rg, ^276^Mt, ^272^Bh, and ^268^Db (bars), in comparison with the lifetime probability curves (dash-dotted lines) calculated for the recently published half-lives of these isotopes (Oganessian et al., 2022; Oganessian et al., 2022b). Yellow denotes α-decay, green denotes spontaneous fission.

Based on the decay properties along the long ^288^Mc decay chain (Figure 3A), four out of eighteen chains were attributed to originate from ^288^Mc (*cf.* Table 1). The missing observation of ^288^Mc in the other fourteen chains is attributed to the decay losses of the short-lived nucleus ^288^Mc ( $T_{1/2}=193_{-13}^{+15}$ ms) during the transport of the ^288^Mc atoms into the detector channel. The observed ratio of the number of detected decay chains starting with ^288^Mc decay to that of chains starting with ^284^Nh (T_1/2_ = $0.90_{-0.06}^{+0.07}$ s) allows a rough estimate of the mean flush-out time from the RTC window into miniCOMPACT of about 0.4 s. The transport time corresponds to approximately two half-lives for ^288^Mc, but it was short enough for the efficient transport of ^284^Nh. Within the observed decay chains, eleven α particles from the decay chain members ^284^Nh, ^280^Rg, ^276^Mt, ^270^Bh, were missing, in agreement with the mean detection efficiency for a single α particle emitted inside the miniCOMPACT detector channel of about 80%. The search for the α decay of the long-lived ^268^Db was performed within the energy interval (7.7–8.0) MeV and within the time interval between the last member of the α-decay chain occurring during the time interval of 200 s and the concluding SF event. Several candidates for α decay of ^268^Db were found (Table 1). However, this search was hindered in some cases by a non-zero background in this energy range, especially from non-volatile byproducts within the first centimeters of the miniCOMPACT detector. The measured time intervals between neighbouring members of all registered decay chains show a very good agreement with the known half-lives, deduced from the observation of about two hundred chains in physics experiments (Oganessian et al., 2013; Rudolph et al., 2013; Gates et al., 2015; Oganessian et al., 2022b). The experimental distributions of correlation times for the decay chain members ^284^Nh, ^280^Rg, ^276^Mt, ^272^Bh and ^268^Db are shown in Figure 4, in comparison with lifetime probability curves calculated for the recently published most precise half-lives (Oganessian et al., 2022; Oganessian et al., 2022b).

Five SF events, which were observed without position and time correlation to any α-decaying precursors within the energy range (9.0–10.5) MeV and a time interval of 1000 s. They could not be assigned to any registered long decay chain from ^288^Mc or ^284^Nh. Two such SF events were registered in the first centimeter of the miniCOMPACT array, and three at the 4th, 6th and 10th centimeter. In four cases, both fission fragments were detected. These SF-only events were considered as real ones and could be attributed to SF branches in early chain members (Oganessian et al., 2022; Forsberg et al., 2016; Rudolph, 2022). A detailed analysis on decay scenario aspects along the decay chain from ^288^Mc will be published elsewhere^2^. Even though the spatial distribution of these SF events in the detector channel resembles the distribution of decay chains from ^284^Nh, the ambiguity in the assignment of these SF-only events did not allow including them for the evaluation of the chemical behaviour.

A comparison of the number of events registered in the present experiment to the number of decay chains from the Mc decay spectroscopy experiment at TASCA (Rudolph et al., 2013) normalized to the number of produced ^288^Mc nuclei reveals that roughly 40% of the reaction products guided to the TASCA focal plane were extracted into the gas chromatography channel. The losses were primarily caused by adsorption of non-volatile species on the RTC surface before reaching the detection setup. In addition, decay losses also significantly contributed to the overall losses of the short-lived ^288^Mc.

### 3.2 Distribution of Mc and Nh in the detection setup

**FIGURE 5:**
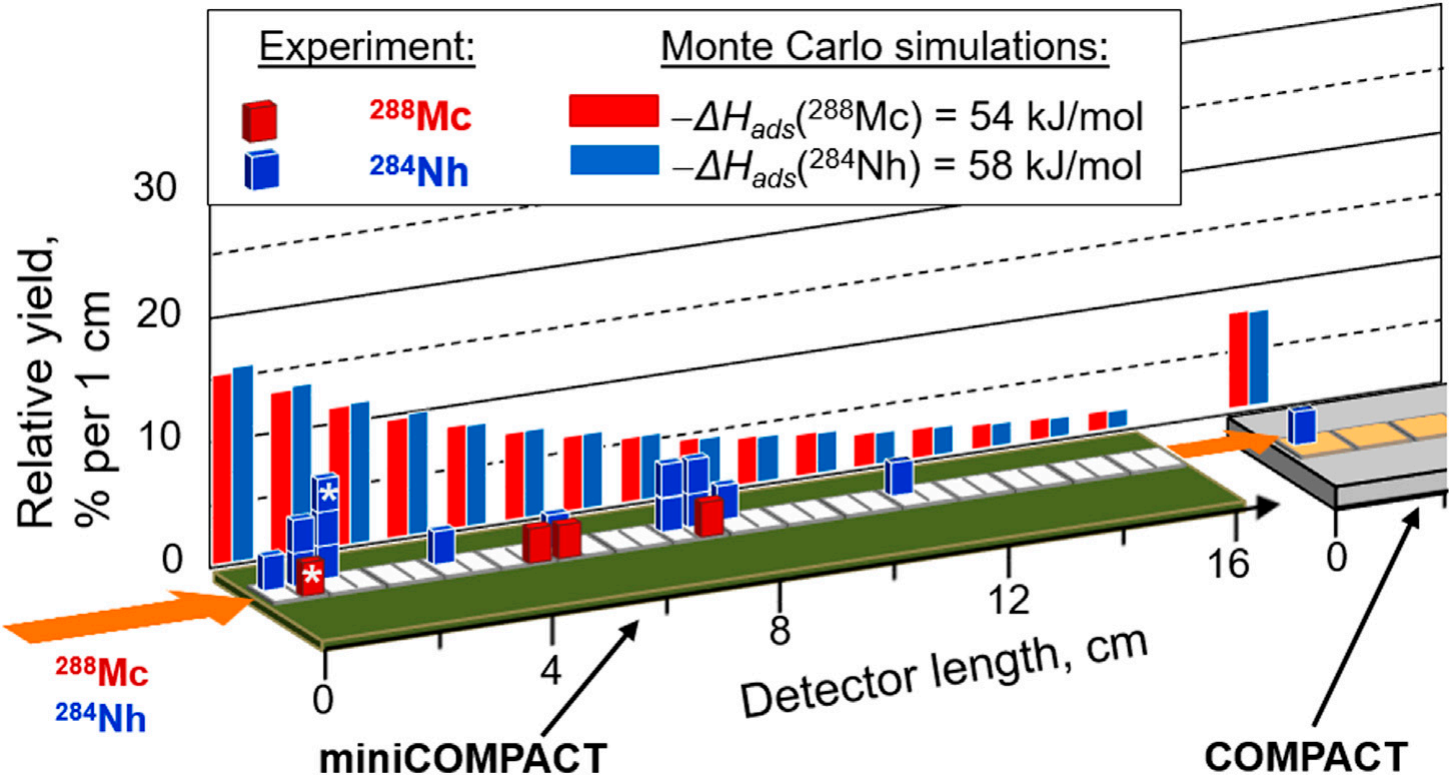
Event distribution in the detection setup. The positions of all observed decay chains in the detection setup assigned to ^288^Mc (red bars) and ^284^Nh (blue bars) are shown together with results of Monte Carlo simulations for the most probable values of the adsorption enthalpy on SiO_2_, −Δ*H*_ads_ (Mc) = 54 kJ/mol and −Δ*H*_ads_ (Nh) = 58 kJ/mol, for Mc and Nh, respectively. According to simulations, a fraction of about 8% of both, Mc and Nh atoms, entering the miniCOMPACT pass through. This fraction is indicated as deposition in the first centimeter of COMPACT. In one decay chain, the observed decays from ^288^Mc and ^284^Nh were distributed over two detectors; their positions are marked with an asterisk.

All observed decay chains originating from ^288^Mc or ^284^Nh were found in the miniCOMPACT detector, except for one decay chain from ^284^Nh, which was registered in the first centimeter of the Au-covered COMPACT. Thus, while all four Mc atoms adsorbed on the SiO_2_ surface, one of the 14 Nh atoms passed the miniCOMPACT detector and a 30-cm long connecting tube made of PTFE between miniCOMPACT and COMPACT, but adsorbed on the very first Au-covered detector. This observation may suggest a stronger interaction with the Au surface compared to interaction with SiO_2_ and PTFE surfaces, in line with the predictions (Pershina et al., 2021). This encourages future studies with higher statistics. The positions of the observed decay chains starting with ^288^Mc and starting with ^284^Nh in the detector channel are presented in Figure 5. All four events originating from ^288^Mc and about 85% of the ^284^Nh events were registered in the first half of the SiO_2_-covered miniCOMPACT detector.

## 4 Discussion

Atoms without retention on the surface would pass miniCOMPACT within about 20 ms. Thus, almost all Nh and Mc nuclei would pass the miniCOMPACT detector array without decay. Clearly, the observation of the decay of Nh and Mc nuclei in the miniCOMPACT detector array demonstrated retention due to adsorption of Nh and Mc on SiO_2_ at room temperature. A key question is in which chemical form Mc and Nh deposited on the SiO_2_ detector surface. Despite the short lifetime of Mc and Nh, the possibility to form compounds through rare interactions with gas impurities cannot be excluded. The main gas impurities, O_2_ and H_2_O, were kept at a ppm level at most, similarly to the previous experiments at TASCA (Yakushev et al., 2014; Yakushev et al., 2022). To form compounds (oxides or hydroxides) in reactions with traces of O_2_ and H_2_O, Mc ions or atoms should be able to break rather strong chemical bonds in the O_2_ and H_2_O molecules after their thermalization in the gas. Energies of selected reactions of the M atoms and M+ ions with the O_2_ and H_2_O molecules, where M is a superheavy element Mc or Nh, or their nearest homologues Bi or Tl, were calculated with the ADF BAND code and are presented in Table 2. All values are positive, ranging from 2.07 to 11.77 eV. Because of the rather high positive values of the heat of reaction, these interactions are not energetically favored at room temperature for Mc and Nh and neither for their homologues Bi and Tl. Thus, the thermodynamical analysis of possible reactions with trace gas impurities clearly suggests that Mc and Nh should stay in the elemental form before they adsorb on the SiO_2_ surface. In order to obtain more quantitative information on the interaction strength between Mc and Nh atoms and the SiO_2_ surface, the Monte Carlo simulation method was applied to obtain numerical information on the interaction strength between SHE species and a surface. This approach is based on the model of mobile adsorption (Zvara, 1985) and allows to simulate individual histories of a large number of single atoms (e.g., 10^6^) migrating through the chromatography column, using actual experimental parameters including the dimension of the column, the gas composition and flow rate, the radionuclide’s half-life etc. Single Mc or Nh atoms moving with the carrier gas along the detector arrays experience many collisions with the surface. In each surface collision, the adsorbed atom is immobilized at the surface for a time τa, which depends on the (experimentally known) oscillation period $\tau_{0}$ , the activation energy needed for its desorption from the surface (*E*_des_), which is a free parameter, and on the temperature, according to the Frenkel’s equation (Frenkel, 1948) (Equation 1).

$$\tau_{a}=\tau_{0}\cdot \exp\left(\left(\frac{E_{des}}{RT}\right)\right)$$ (1)

**TABLE 2:** Calculated values of the energy of reaction for selected chemical reactions of metal M (M = Bi, Mc) with oxygen and water. The initial metallic species are neutral atoms (M) or monovalent cations (M(+)). The initial oxygen and water are molecular O_2_ and H_2_O.

Chemical reaction	Energy of reaction, eV			
	M = Tl	M = Bi	M = Nh	M = Mc
M(+) + O_2_ = MO(+) + O	5.928	2.666	5.705	5.697
M(+) + H_2_O = MO(+) + H_2_	5.431	2.168	5.207	5.200
M(+) + H_2_O = M(OH) + H (+)	9.448	8.613	9.547	10.200
M + O_2_ = MO(+) + O (−)	10.977	8.928	11.765	10.320
M + H_2_O = M(OH) + H	2.065	2.444	5.073	2.393

Supposed that the energy needed for the desorption is equal to the negative adsorption enthalpy, $E_{\text{des}}=-∆H_{\text{ads}}^{{\text{SiO}}_{2}}$ , such simulations were performed for the adsorption of Mc and Nh atoms on SiO_2_ resulting in simulated distributions of Mc and Nh for different $-∆H_{\text{ads}}^{{\text{SiO}}_{2}}$ values. The statistically analysed agreement between the experimental distributions of ^288^Mc and ^284^Nh in miniCOMPACT and the simulated ones enabled deducing the adsorption enthalpy values of Mc and Nh atoms on the SiO_2_ surface, ( $-∆H_{\text{ads}}^{{\text{SiO}}_{2}}$ ), to ${\text{54 }}_{‐5}^{+11}$ kJ/mol for Mc and ${\text{58 }}_{‐3}^{+8}$ kJ/mol for Nh. The distribution of Mc and Nh atoms in miniCOMPACT for their most probable $-∆H_{\text{ads}}^{{\text{SiO}}_{2}}$ values are depicted in Figure 3. The lower and upper limits for these values were determined for a confidence interval (c.i.) of 68% by statistical analysis employing a method of c.i. calculation for experiments with small event numbers (Brüchle, 2003). The obtained −Δ*H*_ads_ values for Mc and Nh on the SiO_2_ surface agree with the theoretically predicted *E*_ads_ values for elemental Nh and Mc on quartz (Pershina et al., 2021), supporting the assignment of Mc and Nh deposition in elemental form.

**FIGURE 6:**
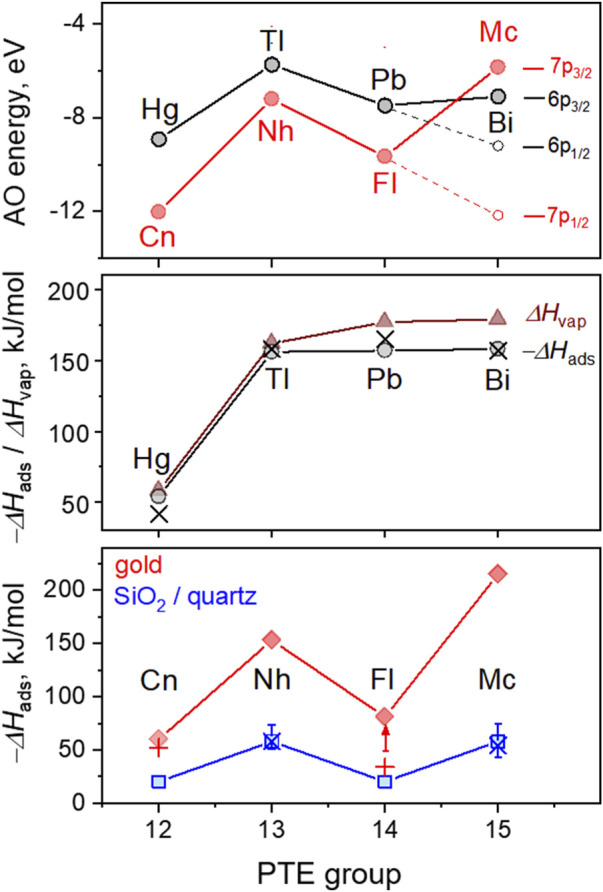
Calculated energies of the highest occupied atomic orbitals for elements of the groups 12–15 of the 6th and 7th period; adsorption enthalpies (−Δ*H*_ads_) on quartz (theoretical values of 6p and 7p elements and experimental values of 6p elements); −Δ*H*_ads_ values on the SiO_2_ (experimental values of 7p elements) and Au surfaces (theoretical and experimental values of 7p elements); and vaporization enthalpies (Δ*H*_vap_). Upper panel: calculated Dirac-Fock energies of the highest occupied AO (in eV) are shown with red symbols for Cn to Mc and with black ones for their homologues, Hg to Bi (Desclaux, 1973). The black and red arrows show the values of the spin-orbit splitting for the 6p and 7p shells, respectively. Middle panel: The calculated and experimental values of −Δ*H*_ads_ on quartz for SHE homologues, Hg to Bi, are shown as grey circles and black crosses, respectively (Pershina, 2018; Trombach et al., 2019; Serov et al., 2013; Steinegger et al., 2016; Düllmann et al., 2022; Kraus, 2021). The experimental values of Δ*H*_vap_ are shown as brown solid triangles (Zhang et al., 2011). Lower panel: calculated values of −Δ*H*_ads_ on quartz and Au for Cn to Mc are shown with blue square and red diamond symbols, respectively (Pershina, 2018; Pershina et al., 2021; Trombach et al., 2019; Düllmann et al., 2022). Experimental −Δ*H*_ads_ on Au for Cn and Fl are shown as red crosses and an arrow in the case of a limit (Eichler et al., 2010; Yakushev et al., 2014; Yakushev et al., 2022). Experimental values from this work, $-∆H_{\text{ads}}^{{\text{SiO}}_{2}}$ (Mc) = ${\text{54 }}_{‐5}^{+11}$ kJ/mol and $-∆H_{\text{ads}}^{{\text{SiO}}_{2}}$ (Nh) = ${\text{58 }}_{‐3}^{+8}$ kJ/mol, are shown by blue crosses with error bars. All panels: the lines connecting the data points are shown to guide the eye. All −Δ*H*_ads_ values are given in kJ/mol.

Theoretical and experimental thermodynamic values for the reactivity and volatility of the superheavy elements in groups 12–15 and their nearest lighter homologues are summarized in Figure 6. For the heaviest members of the groups 12–15 of the PTE, trends in AO energies of the highest occupied orbitals show an irregular course with minima for groups 12 and 14. The spread is significantly more pronounced for SHE compared with their lighter homologues due to the strong spin-orbit splitting in the 7p shell and stabilisation of the 7s and 7p_1/2_ (sub) shells in Cn and Fl, respectively (Figure 6, upper panel). This trend also determines the chemical behaviour, e.g., the adsorption strength of these elements on the gold and quartz surfaces. The calculated and experimental values of −Δ*H*_ads_ on quartz for elements of the 6th row are shown in Figure 6 (middle panel) in comparison with their heat of vaporization (Δ*H*_vap_) values.

The trend in −Δ*H*_ads_ values for the 6p elements is indicative of a strong increase in the interaction strength with the surface from Hg to Tl that remains almost unchanged for Tl, Pb, and Bi (Pershina, 2018; Trombach et al., 2019; Düllmann et al., 2022). The theoretical −Δ*H*_ads_ values show a good agreement with the experimental ones (Serov et al., 2013; Steinegger et al., 2016; Kraus, 2021). The experimental −Δ*H*_ads_ value for Bi was obtained in recent off-line gas chromatography studies with Po and At isotopes at the U-120 M cyclotron in Řež, Czech Republic, and will be published elsewhere. The Δ*H*_vap_ values for these elements follow a similar trend (Zhang et al., 2011). For the heaviest members of groups 12–15, the calculated and experimental −Δ*H*_ads_ values on quartz and Au are shown in Figure 6 (lower panel). The predicted −Δ*H*_ads_ values for adsorption of Nh and Mc on quartz are significantly lower than those of their lighter homologues, Tl and Bi, respectively. Moreover, the trend of the −Δ*H*_ads_ values of the superheavy elements Cn to Mc has also an irregular course, similar to that of the energies of their highest occupied AOs. This difference in the trends between the 6p and 7p elements is attributed to the larger spin-orbital effects on the 7p AO (Figure 6, upper panel) and the relativistic stabilization of the 7p_1/2_ orbital, and is in line with the reduced chemical reactivity in Fl (Schwerdtfeger et al., 2020; Pershina, 2015; Eliav et al., 2015).

## 5 Conclusion

Eighteen decay chains originating from ^288^Mc and its decay products were identified upon adsorption on silicon oxide and gold surfaces (Table 1). The decay properties of the registered chain members are in excellent agreement with those obtained in earlier physics studies. Taking into account experimental conditions and results of state-of-the-art theoretical predictions, we conclude that Nh and Mc were deposited on the silicon oxide surface in the elemental state. The thermodynamic quantities for Nh and Mc, namely, $-∆H_{\text{ads}}^{{\text{SiO}}_{2}}\left(\text{Mc}\right)=54_{-5}^{+11}$ kJ/mol and $-∆H_{\text{ads}}^{{\text{SiO}}_{2}}\left(\text{Nh}\right)=58_{-3}^{+8}$ kJ/mol (68% c.i.), were deduced by using Monte Carlo simulations. Our experimental observations indicate a stronger chemical interaction of Nh and Mc compared to their neighbours Cn and Fl, in accordance with theoretical predictions. The obtained values are significantly (about 100 kJ/mol) lower than those of their lighter homologues, Tl (Serov et al., 2013; Steinegger et al., 2016) and Bi. Thus, the lowest chemical reactivity in groups 13 and 15 is observed for the 7p elements Nh and Mc. This is due to strong relativistic effects, i.e., the large energy splitting in the 7p shell and the stabilization of the 7p_1/2_^2^ subshell has been confirmed experimentally. This work opens the door to future chemical research with even heavier elements, Lv and Ts, to shed light on the evolution of relativistic effects as the 7p_3/2_ subshell is being filled. However, a fast and efficient extraction from a gas-filled chamber to a chemistry setup is required for future SHE experiments beyond Mc to minimize the transport time of very short-lived products to a chemistry apparatus down to a few milliseconds. Recent numerical and experimental results show promise for such chemistry experiments, if a chemistry setup is coupled to a buffer gas cell, in which ions are carried to a chemistry device by electrical fields (Varentsov and Yakushev, 2019; Götz et al., 2021).

## Data Availability

The raw data supporting the conclusions of this article will be made available by the authors, without undue reservation.

## Footnotes

1 Dietzel, D., Yakushev, A., Düllmann, Ch. E., Khuyagbaatar, J., Krier, J., Jäger, E., et al. (2024). Off-line single-atom gas chromatographic adsorption studies of ^211^Bi on SiO_2_ surfaces in the novel miniCOMPACT setup. *Submitt. Radiochim. Acta*. [under revision].

2 Khuyagbaatar, J., Yakushev, A., Düllmann, Ch. E., and Mošať, P. (2024).
